# Implementation of Active Surveillance for Prostate Cancer: A Story of Little Pride and Much Prejudice in the Greek Health Care System

**DOI:** 10.7759/cureus.39330

**Published:** 2023-05-22

**Authors:** Filippos Kapogiannis

**Affiliations:** 1 Urology, Hippokration General Hospital, Athens, GRC

**Keywords:** covid-19, greece, prostate cancer, active surveillance, global healthcare systems

## Abstract

The healthcare system in Greece consists of a mixed public and private sector that contributes to varying extents to the provision of general or specialized health services. Despite intertemporal efforts and investments by the government, the health system remained predominantly underdeveloped in comparison with most European countries. An accurate mirror of the imbalances in cancer care is the underutilization of active surveillance (AS) for prostate cancer. Although AS (a monitoring method to delay or even avoid unnecessary treatment) is becoming the de facto standard of care for low-risk prostate cancer, it remains unpopular in some countries. Focusing on efforts to expand knowledge among the urological community, continuous patient education, and quality improvement of health services will eventually boost national awareness and compliance and promote a radical change of attitude towards AS.

## Editorial

Healthcare in Greece consists of a universal healthcare system provided through national health insurance by the National Healthcare Service (ESY in Greek) and private healthcare. Historically, the public system’s prosperity through the first decade since its innovative establishment in 1983 was followed gradually by a downward path that has led to an outdated health system with structural weaknesses that is far from being described as modern and efficient. The recent economic crisis followed by years of recession and austerity measures imposed on the Greek population had a great impact on every aspect of life, a fact that has led Amnesty International to conclude that Greece is in violation of the right to the enjoyment of the highest attainable standard of physical and mental health in a report from 2020 [1]. To add insult to injury, the unexpected and subsequent COVID-19 pandemic exacerbated preexisting challenges and inherent imbalances and pushed the vast majority of both healthcare providers and recipients to their limits. Since 2010, Greece has undergone extensive and comprehensive healthcare sector reform. The accretive reforms included macroeconomic policy changes (price controls, budget caps) and structural reforms (gatekeeping, e-prescription). As a consequence, the efficacy of ESY has improved, but there is still work to be done in order to ensure that it reaches its full potential. A reflection of the systems’ inadequacy is the active surveillance (AS) implementation efforts regarding prostate cancer (PCa), which unfortunately remain at an infancy stage.

The initial enthusiasm for prostate-specific antigen (PSA) blood test introduction in the early 1990s as a trusted and clinically useful biomarker used for diagnosis and screening rested upon a marked reduction of the proportion of patients who had advanced disease at diagnosis and the age-adjusted prostate cancer mortality as demonstrated in large, prospective, randomized clinical trials [2]. As a result, many countries have adopted the PSA test as a screening tool since the mid-90s, but its limited specificity proved to be a weak point that generated controversies among urological communities and national policy groups. The main objection was the fact that many patients would be diagnosed with indolent screen-detected tumors (i.e., cancer that would otherwise not become clinically manifested over a patient’s lifetime or not result in cancer-related death). Definite treatment offered to them with either radical prostatectomy or radiotherapy would coincide with complications (urinary incontinence, erectile dysfunction) or toxicities (genitourinary, bowel) that could severely impact the quality of life and result in high regret rates [3].

In an effort to counterbalance the possible overdiagnosis and overtreatment of the PSA era, AS was introduced 20 years ago as an alternative approach and is currently considered by various international guidelines as the preferred treatment option for men with low-risk disease and a life expectancy of > 10 years. AS is a management option with curative intent that aims to minimize, postpone, or omit treatment-related side effects without compromising survival. It includes a combination of PSA testing, digital rectal examinations, imaging, and prostate biopsies to monitor prostate cancer at predefined time intervals. Although there are several questions about AS that remain unanswered, its limited adoption, both nationally and internationally, appears to be unjustified given the robust data available in the literature [4].

Acceptance and utilization of AS are hindered by various doctor-, patient-, or healthcare system-related limitations. Physicians play perhaps the most crucial role in the decision-making process. Numerous other factors linked to physicians have been identified as barriers, such as older age, financial incentives, academic or community background, medicolegal constraints, and a lack of high-quality scientific evidence. It is common knowledge that surgeons (not only urologists) in Greece traditionally carry a "surgical culture" that favors treatment against active surveillance and thus exerts a major influence on their patients' final judgment. In addition, the "C" word invokes fear and confusion, and no matter how reassuring a doctor can be, the next step in people's minds is getting a second opinion, which most of the time involves an invasive treatment. Patients, on the other hand, have to cope with the anxiety of living with cancer and, on top of that, undergo a series of follow-up tests, some of which are invasive and potentially harmful. Even those who are not initially reluctant choose to discontinue AS after a couple of years out of fear of missing a window of opportunity for a cure. Lastly, but equally important, the lack of equality and equity throughout the healthcare system forces people who seek cancer care to hit barriers at every turn. More specifically, income, education, geographical location, and discrimination based on ethnicity, race, gender, and disabilities are just a few of the factors that can negatively impact AS. In addition, the absence of organized multidisciplinary service at the national level, an inadequate referral system, and a primitive electronic medical record system simply open the cancer care gap.

One would expect that the COVID-19 pandemic would be an ideal period of time to improve rates of AS and expand its adoption owing to supply restrictions (e.g., cancellation of elective care, redistributing cancer services to COVID-19 ones) or accessibility barriers (lockdowns, travel restrictions), but that just did not happen. In a cross-sectional study conducted in our department between June 2020 and June 2022, which comprised structured questionnaires and interviews with urologists, AS rates remained proportionally very low, if not absent, despite anticipation of a contrary effect. To our disappointment, Greeks could not follow the pace of increasing uptake of AS during the last few years as seen in other countries.

Improvements in prostate imaging, biomarker discovery, and genetic profiling of prostate cancers will very likely change our approach toward AS. Steps forward have been made. Recently, the Hellenic Urological Association developed the so-called "patient office" to support and empower the protection of health service recipients’ rights and strengthen their participation in health policy-making and priority-setting. Nonetheless, there is a need for a more coherent, integrated, and comprehensive reform strategy to cover the unmet health needs of the general population. This multidimensional approach will eventually swing the pendulum on the treatment of low-risk prostate cancer the other way and hopefully remind us of the ancient dictum of medical ethics, "primum non nocere"-first, do no harm!
